# Supplementary material for: Filter Papers to Collect Blood Samples from Dogs: An Easier Way to Monitor the Mass Vaccination Campaigns against Rabies?
Source: Viruses. 2022 Mar 29;14(4):711. doi: 10.3390/v14040711 (PMC9029112; doi:10.3390/v14040711)
Supplement: Supplementary file 1 [file viruses-14-00711-s001.zip › viruses-1596251-supplementary.pdf]

Table S1 : sera titrated using the BioPro ELISA kit versus sera titrated using the FAVN test

Assay	Result	BioProRabies ELISA Ab kit – sera					
		Dogs- Experimental conditions (France)			Dogs- Field conditions (Tunisia)		
		Positive (≥70%)	Negative (<70%)	*Overall agreement*	Positive (≥70%)	Negative (<70%)	*Overall agreement*
FAVN test – sera	Positive (≥0.5 IU/mL)	63	1		39	8	
	Negative (<0.5 IU/mL)	16	53	*87.2%*	7	29	*81.9%*
	Total	79	54		46	37	
